# Important risk factors for gallstones after laparoscopic gastrectomy: a retrospective study

**DOI:** 10.1186/s12893-021-01458-y

**Published:** 2022-01-08

**Authors:** Shohei Fujita, Masaru Kimata, Kenji Matsumoto, Yuichi Sasakura, Toshiaki Terauchi, Junji Furukawa, Yoshiro Ogata, Kenji Kobayashi, Hiroharu Shinozaki

**Affiliations:** Department of Surgery, Saiseikai Utsunomiya Hospital, 911-1, Takebayashi-Machi, Utsunomiya-Shi, Tochigi 321-0974 Japan

**Keywords:** Gallstone disease, Gastric cancer surgery, Laparoscopic surgery

## Abstract

**Background:**

The frequency of gallstones is higher in patients who have undergone gastrectomy than in the general population. While there have been some studies of gallstone formation after open gastrectomy, there are few reports of gallstones after laparoscopic gastrectomy (LG). Therefore, this study aimed to evaluate the incidence of gallstones after LG.

**Methods:**

We retrospectively reviewed the records of 184 patients who underwent LG between January 2011 and May 2016 at Saiseikai Utsunomiya Hospital. After gastrectomy, abdominal ultrasonography was generally performed every 6 months for 5 years. Patients who underwent cholecystectomy before LG, underwent simultaneous cholecystectomy, and did not undergo abdominal ultrasonography, with an observation period of < 24 months, were excluded from the study. Finally, 90 patients were analyzed. Laparoscopic cholecystectomy was performed whenever biliary complications occurred. Patient characteristics were compared using the two-tailed Fisher’s exact test or Chi-square test. In addition, the risk factors for postoperative gallstones were analyzed using logistic regression analysis.

**Results:**

Among the 90 patients included in this study, 60 were men (78%), and the mean age was 65.5 years. Laparoscopic total gastrectomy was performed for 15 patients and laparoscopic distal gastrectomy for 75 patients. D2 lymph node dissection was performed for 8 patients (9%), whereas 68 patients underwent LG with Roux-en-Y reconstruction (76%). Gallstones were detected after LG in 27 of the 90 (30%) patients. Multivariate analysis identified Roux-en-Y reconstruction and male sex as significant risk factors of gallstones after gastrectomy. The incidence of gallstones was significantly higher (53%) in male patients who underwent Roux-en-Y reconstruction. Symptomatic gallstones after laparoscopic cholecystectomy were found in 6 cases (6/27, 22%), and all patients underwent laparoscopic cholecystectomy.

**Conclusion:**

Roux-en-Y reconstruction and male sex were identified as significant risk factors for gallstones after LG.

## Background

Based on low-quality evidence, there is no difference in short-term mortality between laparoscopic gastrectomy (LG) and open gastrectomy, and there is no evidence for any difference in short-term and long-term outcomes. However, the available data are limited, and the confidence intervals were wide, suggesting that the significant benefits or harms of LG cannot be ruled out.

The frequency of gallstones is higher in patients who have undergone gastrectomy than in the general population (6.5–25% vs. 2.2–5.0%) [1–7]. Possible mechanisms associated with this increased incidence include vagotomy, the extent of gastrectomy, method of gastrointestinal reconstruction, and lymph node dissection [1, 8].

Preserving the hepatic branch of vagal nerves reduces the frequency of gallstones [4] In addition, LG can reliably preserve the vagal nerves by a magnification effect [9]; hence, it may reduce the rate of gallstones. While there have been some studies of gallstone formation after open gastrectomy, there are few reports of gallstones after LG. Thus, our retrospective study analyzed the frequencies and risk factors for gallstones in patients who had undergone LG for gastric cancer.

## Materials and methods

We retrospectively reviewed data from 184 patients who underwent LG for gastric cancer at Saiseikai Utsunomiya Hospital between January 2011 and March 2016. All 184 cases involved either total gastrectomy or distal gastrectomy associated with systemic lymph node dissection. The patients had been treated by laparoscopic total gastrectomy (LTG) or laparoscopic distal gastrectomy (LDG) with lymph node dissection and tumor–node–metastasis staging, following the guidelines of the Japanese Classification of Gastric Carcinoma (3rd English edition) and the Japanese Gastric Cancer Treatment Guidelines [10, 11].

In our institution, the LG procedure has been indicated for cT1-2, cN0 gastric cancer. Intestinal reconstruction was performed using the Roux-en-Y method for LTG and Billroth I anastomosis or Roux-en-Y reconstruction for LDG. Our primary reconstructive procedure of choice in LDG was Billroth I. We performed Roux-en-Y for patients with a small remnant stomach or tumor invasion to the duodenum, in which anastomosis was under tension, or for patients with hiatal hernia. The hepatic branch of the vagal nerves was preserved in all patients regardless of the extent of lymph node dissection. We confirmed the vagal nerve’s hepatic branch running across the hepatogastric ligament and then divided this ligament just below the hepatic branch to preserve this nerve. The quality of vagal nerve preservation was confirmed by an expert surgeon who consistently participated in the operation as an operator or assistant. We did not preserve the celiac branch of the vagal nerve irrespective of clinical stage. If the preoperative examination revealed gallbladder disease, we performed simultaneous cholecystectomy.

Abdominal ultrasonography and computed tomography (CT) were generally performed every 6 months for 5 years after the operation. Among the 184 patients, 5 who underwent cholecystectomy before LG, 69 who underwent simultaneous cholecystectomy at the time of LG, 18 who were followed up for less than 24 months, and 2 who did not undergo abdominal ultrasonography were excluded. The remaining 90 patients were analyzed in this study. After LG, if symptomatic gallstones, such as biliary colic, acute cholecystitis, and cholangitis, occurred, cholecystectomy was performed.

### Ethics approval and consent to participate

This retrospective study was approved by the Institutional Review Board (IRB) of Saiseikai Utsunomiya Hospital (IRB No. 2020-17), and all participants provided informed consent.

### Statistical analysis

The statistical analysis was performed with JMP (SAS Institute, Inc., Cary, NC, USA). Patient characteristics were compared using the two-tailed Fisher’s exact test or Chi-square test. The cumulative incidence of gallstones after gastrectomy was evaluated via the Kaplan–Meier method, and differences between the groups were evaluated via the log rank test. The risk factors for postoperative gallstones were analyzed using logistic regression analysis. The factors that were possibly related to gallstone development were estimated using univariate and multivariate analyses. We entered six variables (age, sex, body mass index, type of gastrectomy, extent of dissection, and reconstruction method) into the multivariate regression analysis and then used a stepwise method to select significant variables. p-values of < 0.05 were considered statistically significant.

## Results

**Fig. 1:**
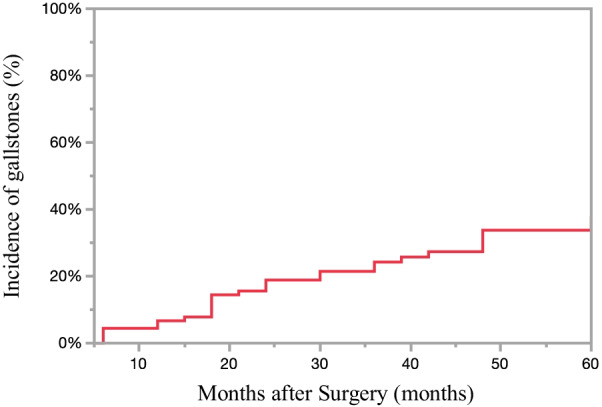
Incidence of gallstones after surgery. Gallstone formation after LG was observed in 27 patients (30%)

Among the 90 patients included in this study, 60 were men (78%), and the mean age was 65.5 years. LTG was performed for 15 patients and LDG for 75 patients. D2 lymph node dissection was performed for 8 patients (9%), whereas 68 patients underwent LG with Roux-en-Y reconstruction (76%). Gallstone formation after LG was observed in 27 patients (30%) in this study (Fig. 1). The median follow-up period between gastrectomy and the diagnosis of gallstones was 22.5 months (range, 6–48 months). The characteristics of the 90 patients by group are shown in Table 1. The median follow-up period after gastrectomy was 46.2 months (range, 24–60). The average time between gastrectomy and diagnosis of gallstones was 24.3 months (range, 6–48). The mean age of the 63 patients (36 men and 27 women) in the stone-negative group was 64.4 years (range, 32–85), and that of the 27 patients (24 men and 3 women) in the stone-positive group was 67.9 years (range, 44–85).

**Table 1:** Characteristics of the gastric cancer patients and the laparoscopic gastrectomy procedures

Variables	Stone-negative n = 63	%	Stone-positive n = 27	%	p-value
Age (years)	64.4 (32–85)		67.9 (44–85)		0.22
Sex (M/F)					
Male	36	60	24	40	
Female	27	90	3	10	0.002
Body mass index (kg/m^2^)	23.2 (12.8–34.7)		24.1 (18.1–29.7)		0.22
Type of gastrectomy					
LTG	9	60	6	40	
LDG	54	72	21	28	0.36
pStage					
I	53	68	25	32	
II	3	75	1	25	
III	1	100	0	0	
IV	0	0	0	0	0.60
Extent of dissection					
D1	15	71	6	29	
D1 +	42	69	19	31	
D2	6	75	2	25	0.83
Reconstruction after LG					
B-I	17	94	1	6	
R-Y	46	64	26	36	0.005
Operation time (min)	287		311		0.069
Blood loss (mL)	43		63		0.083

*LG* laparoscopic gastrectomy, *B-I* Billroth-I, *R-Y* Roux-en-Y

**Fig. 2:**
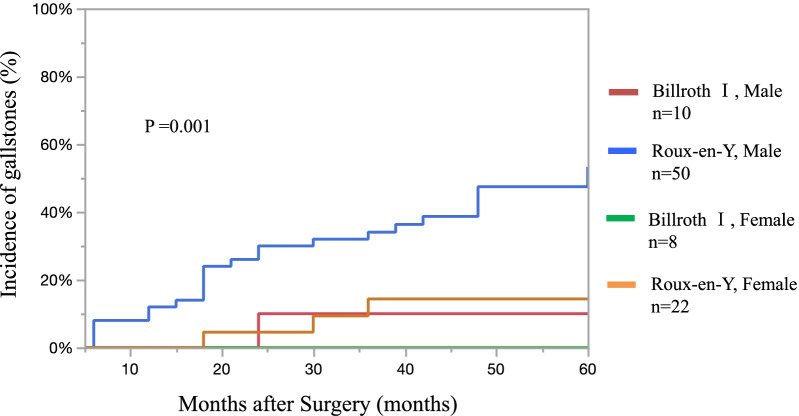
Incidence of gallstones after surgery by reconstruction method and sex. The incidence of gallstones was significantly highest (53%) in male patients who underwent Roux-en-Y reconstruction (p = 0.001)

The findings of risk factor analysis for gallstones after LG are shown in Table 2. Among the 75 patients who underwent LDG, 21 developed gallstones. Moreover, 6 of the 15 patients who underwent LTG developed gallstones (28% vs 40%; p = 0.36). No statistically significant differences were noted in the frequency of gallstone formation between the various types of LG. However, compared to those who underwent Billroth I anastomosis, a significantly higher proportion of patients developed gallstones after Roux-en-Y reconstruction (36% vs 6%; p = 0.010). The proportion of patients with gallstones was higher among men than among women (40% vs 10%; p = 0.003). Further, age, body mass index, and extent of lymph node dissection were not associated with gallstone formation. Multivariate analysis using the stepwise method identified Roux-en-Y reconstruction and male sex as significant risk factors for gallstone formation after LG. The incidence of gallstones was compared between four subgroups, according to the two most significant risk factors (sex and reconstruction method, Fig. 2). The incidence of gallstones was significantly highest (53%) in male patients who underwent Roux-en-Y reconstruction (p = 0.001).

**Table 2:** Univariate and multivariate analysis of risk factors associated with gallstones after laparoscopic gastrectomy

Variables	Stone-negative n = 63	Stone-positive n = 27	Univariate	Multivariate	
			p-value	Odds ratio (95% CI)	p-value
Age (years)					
< 70	38	14			
≥ 70	25	13	0.49		
Sex (M/F)					
Male	36	24			
Female	27	3	0.003	0.174 (0.046–0.655)	0.010
Body mass index (kg/m^2^)					
< 23	32	9			
≥ 23	31	18	0.17		
Type of gastrectomy					
LDG	54	21			
LTG	9	6	0.37		
Extent of dissection					
D1/D1 +	57	25			
D2	6	2	1.00		
Reconstruction after laparoscopic gastrectomy					
Billroth-I	17	1			
Roux-en-Y	46	26	0.010	9.001 (1.04–71.4)	0.040

*LDG* laparoscopic distal gastrectomy, *LTG* laparoscopic total gastrectomy, *CI* confidence interval

Among the 27 cases in whom gallstones were noted, 6 (22%) had gallstone symptoms (Table 3). One patient had biliary colic, three had acute cholecystitis, and two had common bile duct stones and cholangitis. The reconstruction method for these six patients was Roux-en-Y. Regarding acute cholecystitis management, percutaneous transhepatic gallbladder drainage was performed in one patient. Endoscopic retrograde cholangiopancreatography (ERCP) and endoscopic sphincterotomy using an enteroscope were performed in two patients with common bile duct stones and cholangitis. All six patients underwent laparoscopic cholecystectomy.

**Table 3:** Treatment of symptomatic gallstones after laparoscopic gastrectomy

Symptoms	n = 6	%	Treatment
Biliary colic	1	17	Laparoscopic cholecystectomy
Acute cholecystitis	3	50	Laparoscopic cholecystectomy after PTGBD
CBD stone/cholangitis	2	33	Laparoscopic cholecystectomy after ERCP

*CBD* common bile duct, *PTGBD* percutaneous transhepatic gallbladder drainage, *ERCP* endoscopic retrograde cholangiopancreatography

## Discussion

Gallstone formation after LG was observed in 30% of the patients. Roux-en-Y reconstruction was identified as a significant risk factor for gallstone formation after gastrectomy. A possible reason was that exclusion of the duodenum during reconstruction was associated with gallstone formation. Food passage through the duodenum serves as a stimulus for cholecystokinin secretion, and this hormone causes the contraction of the gallbladder through the humoral regulation system. Therefore, it is postulated that the exclusion of the duodenum leads to changes in the pattern of cholecystokinin secretion, resulting in decreased gallbladder contraction and an increased risk of gallstone formation [1]. In Roux-en-Y reconstruction, the bacterial count in the duodenum has been confirmed to significantly increase because of biliary stasis, dysfunction of the sphincter of Oddi, and hypoacidity in the duodenum. In this state, the incidence of bactibilia affects the formation of gallstones [12].

Furthermore, neurological disorders caused by lymphadenectomy for gastrectomy are considered the reason for gallstone formation; damage to the vagal nerve’s hepatic branch induces a reduction in the contractile function of the gallbladder, which may lead to stagnation of bile juice [4]. Our study considered that the magnification effect due to laparoscopic surgery could reliably preserve the hepatic branch of vagal nerves and reduce the incidence of gallstones. However, despite the preservation of the hepatic branch of vagal nerves, a high incidence of gallstones (30%) was noted. In a study of 10 cadavers, innervation of the gallbladder predominantly occurred through two routes [8]. One was from the anterior hepatic plexus containing the branches arising from the hepatic division of the vagal nerves and the celiac plexus. The other route was from the posterior hepatic plexus, containing the branches originating from the celiac branches of the posterior vagal trunk and the celiac plexus [8]. During fundoplication, cutting the hepatic branch of the anterior vagus nerve may reduce gallbladder size; however, it does not affect the ejection fraction [13]. A study found that the occurrence of gallstones after Roux-en-Y reconstruction following LDG was significantly less common in patients with the preservation of the celiac branch of the vagal nerve than in patients with resection of the celiac branch (16 vs. 33%, p = 0.035) [14]. However, LG with preservation of the celiac branch of the vagal nerve has limited adaptation, and it is not generally performed due to the complexity of the procedure.

Several studies found that the development of gallstone disease is associated with total gastrectomy [12, 15]. In this study, although there were no significant statistical differences in the frequency of gallstone formation between LTG and LDG partly due to the small number of LTG cases (40% vs. 28%; p = 0.36), the incidence of gallstones after LG was rather higher in LTG. However, 20 of the 57 patients who had undergone LDG with Roux-en-Y reconstruction developed gallstones (35%; data not shown), and reconstruction for LTG was performed using Roux-en-Y reconstruction. The frequency of gallstone formation between LTG and LDG with Roux-en-Y reconstruction was almost equivalent (40% vs. 35%). These factors lead us to suggest that the exclusion of the duodenum during reconstruction was associated with gallstone formation, and the various types of LG were not associated.

Although gallstone incidence in the general population is reported to be higher in women than in men, this study found that the incidence of gallstones after gastrectomy was higher in men [16, 17]. Various studies have shown no significant difference in the incidence of gallstones between men and women after LG [15, 17, 18]. Although a study showed that the incidence of gallstones after LG is higher in men, the mechanism underlying this phenomenon remains unclear [19]. The incidence of gallstones in the general population is higher in women than in men because estrogen increases biliary cholesterol secretion, causing cholesterol supersaturation of bile, which enhances cholesterol gallstones. In contrast, the most frequent type of gallstone in patients after LG is pigment stones, which include calcium bilirubinate stones and black stones because bacteribilia combined with vagotomy enhances pigment gallstone formation [12]. The different types and mechanisms of occurrence of gallstones may explain why the incidence of gallstones after LG was higher in men.

The 30% incidence of gallstones is rather high, and it is more likely to be detected by ultrasonography than by CT alone, in which it is difficult to detect gallstones without calcification. In a study that examined the incidence of gallstones by regular ultrasonography, its incidence was reported to be 25.7% [4]. In another study that performed ultrasonography, the incidence of gallstones was reported to be 33% for patients who underwent laparoscopic-assisted distal gastrectomy with resection of the celiac branch of the vagal trunk, which is the same with our operation procedure [14]. Another factor contributing to the high incidence of gallstones is that 76% of the patients underwent Roux-en-Y reconstruction, and 78% of the patients were males, which were significantly more associated with gallstone formation in this study.

Regarding the 27 patients with gallstones, 6 patients (22%) with symptoms of gallstones underwent laparoscopic cholecystectomy. However, Hashimoto et al. [20] performed laparoscopic cholecystectomy in patients with gallstones after gastrectomy, and for 26% of the patients, conversion to laparotomy was performed because of adhesions. If the cholelithiasis falls into the common bile duct, it is crucial to perform ERCP. However, enteroscopy is required in patients who underwent Roux-en-Y reconstruction. The success rate of ERCP for common bile duct stone clearance has been reported to be 81.2% in patients who undergo Billroth I anastomosis and 23.7% in patients who undergo non-Billroth I anastomosis [21]. ERCP failures in those who underwent Roux-en-Y reconstruction were probably a result of the length and sharp angulation of the Roux limb, making it difficult to negotiate the scope’s passage to the papilla [22]; several of these patients were referred for surgical or percutaneous interventions. Laparoscopic surgery for common bile duct stone is not common and is likely to result in laparotomy. Even if LG is performed for gastric cancer, the benefit of small wound size is reduced if laparotomy is performed for subsequent common bile duct stones.

In this study, 22% of patients with symptoms of gallstones underwent laparoscopic cholecystectomy. In another study, 26% of patients who developed gallstones after gastrectomy underwent cholecystectomy during the 5-year follow-up [23]. Since a high percentage of patients who develop gallstones require surgery, it is important to prevent gallstones after gastrectomy to the maximum extent possible. The need to conduct routine prophylactic cholecystectomy, simultaneously with gastrectomy, has been widely discussed but remains controversial [12]. Those who underwent prophylactic cholecystectomy did not experience additional perioperative complications related to biliary surgery. Moreover, no additional time and costs were associated with gastrectomy because of the comparable duration of surgery and the length of postoperative stay [24]. In a randomized controlled trial of 130 patients, 65 underwent prophylactic cholecystectomy, and another 65 underwent standard gastric surgery only for curable cancers; the cholelithiasis-free survival rate did not show statistical significance between the two groups (p = 0.267) [25]. Although the sample size was small, this result showed that prophylactic cholecystectomy was not required for all patients. However, prophylactic cholecystectomy may be considered for patients at a higher risk of cholelithiasis, such as those who have undergone Roux-en-Y reconstruction. Younger patients with early gastric cancer whose life expectancy is high should also be considered for prophylactic cholecystectomy. Surgeons have the moral and legal obligation to adequately explain all treatment-related details to their patients [26]. Until now, the increased incidence of gallstones had not been well informed in preoperative gastrectomy. Surgeons should inform their patients about the increased risk of gallstone formation after LG, especially those who will undergo Roux-en-Y reconstruction, male patients, and younger patients with early gastric cancer whose life expectancy is high. Therefore, we recommend prophylactic cholecystectomy as an acceptable procedure for patients who would prefer it, particularly the abovementioned patients. In a randomized clinical trial involving 521 adults, the use of 300 or 600 mg of ursodeoxycholic acid, compared to the use of a placebo, resulted in a significantly decreased proportion of patients who developed gallstones within 12 months after gastrectomy (5.3% in the 300 mg group, 4.3% in the 600 mg group, and 16.7% in the placebo group) [27]. These findings suggest that ursodeoxycholic acid administration may be considered for high-risk patients after gastrectomy.

The limitations of this study require consideration. This was a single-center retrospective study with a small sample population. Further, there was no comparison between patients who underwent LG and those who underwent open gastrectomy. In our hospital, open gastrectomy is not performed by the same surgeon who performs LG; hence, preservation of the hepatic branch of vagal nerves could not be determined with only operation records, thus making it difficult to make a simple comparison. In studies of 17 325 patients (laparoscopy, 678, vs. open 16647) and 1284 patients (laparoscopy, 980, vs. open, 304), there were no significant differences between laparoscopic and open gastrectomy [15, 19]. A prospective investigation with a larger number of patients is warranted to clarify the significance of prophylactic cholecystectomy.

In conclusion, Roux-en-Y reconstruction and male sex ware identified as a significant risk factors for gallstone formation after LG.

## Data Availability

The datasets used and analysed during the current study are available from the corresponding author on reasonable request.

## Abbreviations

ERCP: Endoscopic retrograde cholangiopancreatography
LDG: Laparoscopic distal gastrectomy
LG: Laparoscopic gastrectomy
LTG: Laparoscopic total gastrectomy
